# Evaluation of two MALDI-TOF MS systems and extraction methods for identification of filamentous fungi recovered from clinical specimens

**DOI:** 10.1128/jcm.01548-24

**Published:** 2025-01-14

**Authors:** Eric M. Ransom, Meghan A. Wallace, Nathan P. Wiederhold, Connie Cañete-Gibas, Carey-Ann D. Burnham

**Affiliations:** 1 Department of Pathology, Case Western Reserve University School of Medicine 12304 , Cleveland, Ohio, USA; 2 Department of Pathology and Immunology, Washington University in St. Louis School of Medicine 12275 , St. Louis, Missouri, USA; 3 Fungus Testing Laboratory, Department of Pathology and Laboratory Medicine, The University of Texas Health Science Center at San Antonio 14742 , San Antonio, Texas, USA; 4 Department of Molecular Microbiology, Pediatrics, and Medicine, Washington University in St. Louis School of Medicine 12275 , St. Louis, Missouri, USA; University of Utah, Salt Lake, Utah, USA

**Keywords:** MALDI, fungal identification, mycology, Bruker, BioTyper, bioMérieux, VITEK MS, Mold, Mould

## Abstract

Rapid and accurate identification of cultured molds is important to determine clinical significance and therapeutic decision-making. Conventional mold identification uses phenotypic macroscopic and microscopic characterization; however, this can take days or weeks for colony maturity and definitive microscopic structure formation, be limited to genus-level identification, and be misidentified due to morphologic mimics or similarities between closely related species. Matrix-assisted laser desorption ionization–time of flight mass spectrometry (MALDI–TOF MS) revolutionized bacterial and yeast identification but remains uncommon for molds in part because of limited reference libraries. Here, a retrospective 5-year review at a large teaching hospital found that 88.6% of identified molds were in the Bruker Filamentous Fungi Library 3.0 and 91.5% in the VITEK Knowledge Base Library 3.2.0. A prospective evaluation was also performed on early growth from 205 consecutive, working clinical isolates. Each mold was processed using the VITEK chemical extraction method and modified NIH chemical plus bead-beating extraction method; both extractions were tested on both systems. When compared to conventional identification, more molds were identified using VITEK extractions over NIH extractions using the VITEK (65 and 59%) and Bruker (56 and 54%) systems, respectively, using the ≥1.5 log Bruker threshold. VITEK MS identified more molds, regardless of the extraction method. Isolates without consensus agreement (*n* = 116) underwent sequence-based identification, which demonstrated that conventional identification had the highest genus-level (84%) but lowest species-level (3%) identification rates compared to VITEK (59 and 52%, respectively) and Bruker (52 and 36%) using VITEK extractions. Taken together, our findings suggest both MALDI-TOF systems can supplement conventional mold identification to optimize identification rates with species-level distinction.

**IMPORTANCE:**

Mold identification using matrix-assisted laser desorption ionization–time of flight mass spectrometry (MALDI-TOF MS) remains uncommon in clinical laboratories. Contributing concerns include limited genus/species spectra in the MALDI-TOF MS libraries, varying success rates in the literature regarding extraction methods and instrumentation, and the lack of practical performance evaluations using early mold colony growth, which would be used in a clinical mycology laboratory. This study used multiple approaches to improve our understanding of the clinical utility and performance of MALDI-TOF MS mold identification.

## INTRODUCTION

Filamentous fungi (molds) are commonly found in the environment but can also be important human pathogens. Infections can be associated with significant morbidity and mortality rates, particularly among immunocompromised patients. A clinical mycology laboratory plays a critical role by culturing and identifying molds. To date, the standard-of-care (SOC) identification method for molds is phenotypic macroscopic and microscopic characterization. There is growing evidence that matrix-assisted laser desorption/ionization-time-of-flight mass spectrometry (MALDI-TOF MS) that revolutionized clinical bacteriology can improve mold identification (1–3). Potential benefits of using MALDI-TOF MS for mold identification include improved species-level accuracy, shorter turnaround times, and reduced training period for laboratory staff, which is critical with medical laboratory technologist labor shortages and an aging workforce. Another potential advantage of mold identification by MALDI-TOF MS is mitigating laboratory-acquired infections by identifying molds prior to colony maturation.

Two key factors for successful mold identification by MALDI-TOF MS are the extraction step and the robustness of the spectra database. The extraction process is important for inactivating the infectious mold spores and for improving run spectra. Each MALDI-TOF MS manufacturer has a recommended mold extraction protocol, but several studies have reported modified or alternative approaches with varying success and clinical implementation feasibility (1, 2, 4). The NIH extraction method is one of the more promising methods, which has been shown to offer rapid and accurate results by using silica beads to improve cell lysis (5, 6). After samples are extracted and run on a MALDI-TOF MS system, the generated spectra are compared to a reference database. To date, mold databases are considerably smaller than their bacterial counterparts. There has been concern that mold databases lack sufficient microbial diversity for clinical utility. The two primary MALDI-TOF MS manufacturers, bioMérieux and Bruker, continue to release updated databases with additional mold spectra.

**Fig 1:**
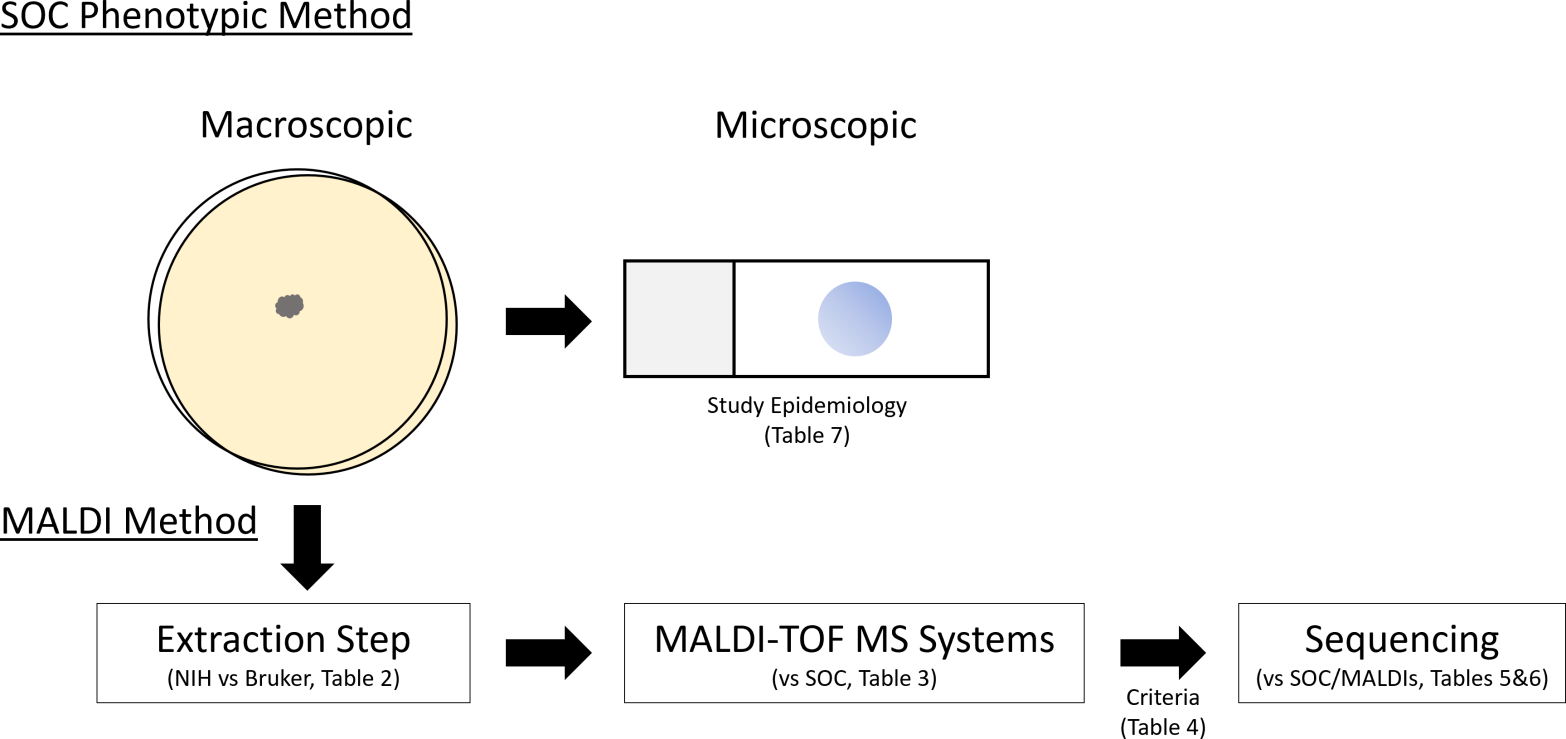
Study overview: experimental design and corresponding result Tables. Molds recovered in culture were identified by standard-of-care (SOC) conventional phenotypic methods (microscopic and macroscopic) and by MALDI-TOF MS. MALDI-TOF MS identification was evaluated with multiple extraction methods and instrumentation systems. Sequence-based identification was performed on a subset of isolates.

Here, we used a retrospective review to determine if these updated databases offer sufficient mold breadth for clinical implementation. In addition, we performed a prospective side-by-side comparison of the two MALDI-TOF MS systems using 205 consecutive clinical isolates to evaluate performance for routine clinical identification (Fig. 1). This comparison also included a side-by-side comparison of different extraction methods to optimize mold identification rates. Because a major objective of this study was to achieve a comparable turnaround time to SOC phenotypic identification, MALDI-TOF MS testing was performed on early growth (<3 days of visible growth) to determine expected performance in a clinical mycology setting.

## MATERIALS AND METHODS

### Retrospective review

The retrospective review consisted of all reported mold identifications from mycology cultures collected June 2015 to June 2020 (*n* = 2,696), including all specimen types. There are more mold identifications than cultures because >1 filamentous mold was recovered from 11.9% of cultures.

### Fungal identification

SOC identification was predominantly phenotypic macroscopic and microscopic identification supplemented with molecular identification upon physician request or to complete the College of American Pathologists’ checklist requirement to confirm the identification of dimorphic fungi. For the prospective study, molds were consecutively recovered from clinical specimens submitted from November 2020 to April 2021 to the microbiology laboratory at the Barnes-Jewish Institute of Health. Repeat mold identifications from the same patient were excluded from this study. Importantly, this study received institutional review board approval and waiver of consent.

### MALDI-TOF MS identification

Upon visualization of a mold on a routine culture medium, molds were subcultured to Sabouraud dextrose agar (Remel, Lenexa, KS) and grown at room temperature (~20–22°C) under atmospheric conditions for MALDI-TOF MS identification. Subculturing was necessary to generate sufficient growth as to not interfere with routine clinical identification practices and to obtain sufficient colony material for two extractions. Extractions of subcultured growth were performed once there was sufficient growth for two extractions and no more than 3 days after visualization. Three extraction methods were investigated. The bioMérieux method was performed using the VITEK MS Mould Kit (bioMérieux), which uses formic acid, acetonitrile, and centrifugation. The standard Bruker method was also performed according to the manufacturer’s instructions. This method similarly uses formic acid, acetonitrile, and centrifugation. The third extraction method (mNIH) published by NIH is a rapid one-step extraction method that also uses zirconia–silica beads (6). All extractions were spotted (1 µL) in duplicate and allowed to dry in a biosafety cabinet. Matrix (1 µL) then was overlaid and dried in a biosafety cabinet. The Bruker MALDI-TOF MS data were generated using automated spectra generation; however, manual spectra acquisition was reflexed to optimize spectra compilation when automated acquisition failed. Per manufacturers’ instructions, quality control and calibration material were required to pass for any data generation. All reagents and materials were used prior to their expiration dates. To display comparable categories for data analyses, VITEK MS result messages 150 and 201 were reclassified as “No Peaks” and “Peaks, but no ID,” respectively.

### Databases

Extracts were run on the VITEK MS and Bruker BioTyper MALDI-TOF MS systems according to manufacturers’ instructions. Spectra were analyzed using the Knowledge Base 3.2.0 and Bruker Filamentous Fungi Library 3.0. Bruker spectra were also analyzed using the Centers for Disease Control and Prevention library, but no additional identifications were found.

### Sequencing

Any isolate without consensus findings across methods was sent for sequencing to the Fungus Testing Laboratory at the University of Texas Health Science Center (San Antonio, TX). Sequencing targets varied by genus and may have included the internal transcribed spacer regions 1/2 (ITS), D1/D2 (domain of 28S), beta-tubulin (*TUB*), translation elongation factor 1α (*TEF*), actin (*ACT*), or glyceraldehyde-3-phosphate dehydrogenase (*GPD*). BLASTn searches of the generated sequences were performed in GenBank. Results were considered significant with an E-value of 0.0, 98–100% identity, and at least 90% query coverage.

### Statistical analyses

Statistical significance was determined in R using a two-sided Fisher’s exact test and a *p-*value limit of 0.05 and using an odds ratio with a *p-*value limit of 0.05.

## RESULTS

### Assessment of MALDI-TOF MS mold libraries

Two commonly used MALDI-TOF MS systems in clinical microbiology laboratories are the Bruker BioTyper and bioMérieux VITEK MS. To determine if the reference spectra databases contain sufficient mold representation for routine clinical use, each system’s mold database was compared to the list of reported molds from our clinical laboratory over a 5-year period. This epidemiological assessment offers a retrospective best-case-scenario approach to evaluate if these databases could have identified previously reported mold isolates. Our laboratory reported 2,696 molds over the 5-year period (Table 1). The most frequently reported mold identification was *Penicillium* species (19.5% of all molds). “Mold” (unable to provide additional specificity) was tied for second, reiterating the frequent inability to identify filamentous fungi to any appreciable level using the SOC phenotypic identification method. Most reported identifications (77.0%) were unable to achieve species-level identification phenotypically, which increased to 82.3% when excluding for molecular confirmation/assistance (e.g., *Histoplasma capsulatum*, *Steccherinum laeticolor*).

**TABLE 1:** Reported fungal identifications from our laboratory and presence of respective molds in the Bruker and VITEK databases^*a*^

Reported identifications	*n*	Percentage of total	Bruker	VITEK
*Penicillium* species	526	19.5	P*	P*
Mold (nonsporulating/sterile)	235	8.7	n/a^*b*^	n/a^*b*^
*Aspergillus fumigatus*	235	8.7	P	P
*Aspergillus* species, not *A. fumigatus* or *A. flavus*	235	8.7	P*	P*
*Trichophyton* species	218	8.1	P*	P*
*Fusarium* species	105	3.9	P*	P*
*Cladosporium* species	88	3.3	P*	P*
*Aspergillus flavus*	83	3.1	P	P
*Aspergillus niger*	74	2.7	P	P
*Alternaria* species	69	2.6	P*	P*
*Paecilomyces* species	65	2.4	A	P*
*Histoplasma capsulatum*	64	2.4	A	P
*Scedosporium* species	59	2.2	P*	P*
Dematiaceous fungus	58	2.2	P*	P*
*Curvularia* species	55	2.0	P*	P*
*Aspergillus terreus*	33	1.2	P	P
*Arthrographis* species	33	1.2	P*	A
*Acremonium* species	32	1.2	P*	P*
*Mucor* species	28	1.0	P*	P*
*Blastomyces dermatitidis/gilchristii*	27	1.0	A	P
*Scopulariopsis* species	22	0.8	P*	A
*Chaetomium* species	19	0.7	P*	P*
*Rhizopus* species	17	0.6	P*	P*
*Microsporum* species	17	0.6	P*	P*
*Epicoccum* species	15	0.6	P*	P*
Dermatophyte	14	0.5	P*	P*
*Menispora* species	12	0.4	A	A
*Exophiala* species	12	0.4	P*	P*
*Rhinocladiella* species	12	0.4	P*	P*
*Pithomyces* species	12	0.4	A	A
*Fusarium solani*	11	0.4	P	P
*Trichoderma* species	11	0.4	P*	P*
*Scedosporium prolificans* (*Lomentospora prolificans*)	10	0.4	P	P
*Syncephalastrum* species	10	0.4	P*	A
*Phoma* species	10	0.4	P	A
*Beauveria* species	9	0.3	P*	P*
*Bipolaris* species	8	0.3	A	A
*Coccidioides posadasii/immitis*	9	0.3	A	P
*Scedosporium apiospermum*	7	0.3	P	P
*Hormographiella* species	7	0.3	A	A
*Aspergillus clavatus*	6	0.2	P	A
*Nigrospora* species	5	0.2	A	A
*Trichophyton rubrum*	5	0.2	P	P
*Apophysomyces* species	4	0.1	A	A
*Exophiala* (*Wangiella*) *dermatitidis*	4	0.1	P	P
*Graphium* species	4	0.1	A	A
*Verticillium* species	4	0.1	A	A
Zygomycete	4	0.1	P*	P*
*Aurantiporus fissilis*	4	0.1	A	A
*Chrysosporium* species	3	0.1	P*	A
*Dactylaria* species	3	0.1	A	A
*Alternaria* (*Ulocladium*) species	3	0.1	A	A
Basidiomycete	3	0.1	P*	P*
*Thyronectria austroamericana*	3	0.1	A	A
*Aureobasidium pullulans*	2	0.1	P	P
*Aureobasidium* species	2	0.1	P*	P*
*Cunninghamella* species	2	0.1	P	A
*Phialophora* species	2	0.1	P*	P*
*Rhizomucor* species	2	0.1	A	A
*Scytalidium* species	2	0.1	P*	A
*Sporothrix schenckii*	2	0.1	P	P
*Trichophyton violaceum*	2	0.1	P	P
*Cladophialophora* species	2	0.1	P*	P*
*Coprinellus* species	2	0.1	A	A
*Humicola phialophoroides*	2	0.1	A	A
*Lasiodiplodia missouriana*	2	0.1	P^	A
*Lasiodiplodia theobromae*	2	0.1	P^	A
*Malbranchea* species	2	0.1	A	A
*Stereum ostrea*	2	0.1	A	A
*Lichtheimia* species (formerly *Absidia* species)	1	0.0	P*	P*
*Aspergillus nidulans*	1	0.0	P*	P*
*Gliocladium* species	1	0.0	A	A
*Scedosporium boydii* (*Pseudoallescheria boydii*)	1	0.0	P	P
*Pseudozyma* species	1	0.0	A	A
*Sporotrichum* species	1	0.0	A	A
*Sporothrix* species	1	0.0	P*	P*
*Stemphylium* species	1	0.0	A	A
*Trichaptum biforme*	1	0.0	A	A
*Acrodontium salmoneum*	1	0.0	A	A
*Arthropsis hispanica*	1	0.0	A	A
*Colletotrichum* species	1	0.0	A	A
*Coniochaeta* (formerly *Lecythophora*) species	1	0.0	P	P
*Coprinellus micaceus*	1	0.0	A	A
*Coprinellus radians*	1	0.0	A	A
*Dentocorticium portoricense*	1	0.0	A	A
*Exophiala bergeri*	1	0.0	A	A
*Gliomastix* species	1	0.0	A	A
*Gymnopilus suberis*	1	0.0	A	A
*Humicola olivacea*	1	0.0	A	A
*Hyphodermella rosae*	1	0.0	A	A
*Irpex lacteus*	1	0.0	A	P
*Cubamyces lactineus* (*Leiotrametes lactinea*)	1	0.0	A	A
*Metarhizium rileyi/Nomuraea anemoniodes*	1	0.0	A	A
*Mortierella alpina*	1	0.0	A	A
*Nigroporus vinosus*	1	0.0	A	A
*Nothophoma quercina*	1	0.0	A	A
*Verruconis gallopava* (*Ochroconis gallopava*)	1	0.0	A	A
*Peniophora* species	1	0.0	A	A
*Pestalotiopsis samarangensis*	1	0.0	A	A
*Phaeoacremonium* species	1	0.0	A	A
*Phialemonium inflatum* (*Paecilomyces inflatus*)	1	0.0	A	A
*Phialemonium* species	1	0.0	A	A
*Physisporinus* species	1	0.0	A	A
*Plectosphaerella cucumerina*	1	0.0	P	A
*Rigidoporus pouzarii*	1	0.0	A	A
*Scedosporium aurantiacum*	1	0.0	P	A
*Schizophyllum radiatum*	1	0.0	A	A
*Schizophyllum* species	1	0.0	A	A
*Steccherinum laeticolor*	1	0.0	A	A
*Talaromyces novojersensis*	1	0.0	P^	A
*Talaromyces rugulosus* (*Penicillium rugulosum*)	1	0.0	P	A
*Tolypocladium* species	1	0.0	A	A
*Trametes* species	1	0.0	A	P*
*Trametes versicolor*	1	0.0	A	A

^*a*^ P: present in database; P*: present and potentially improved species distinction in database; P^: present but only genus distinction in database; A: absent in database.

^*b*^ n/a, not applicable.

**Fig 2:**
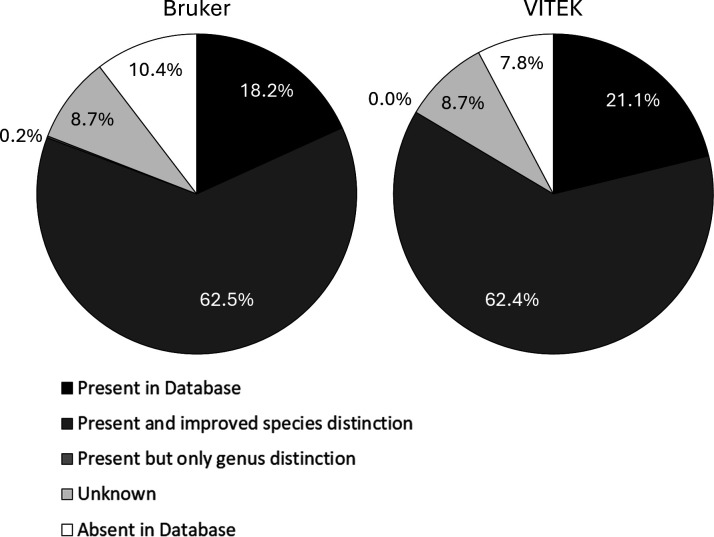
Clinical applicability of MALDI-TOF MS mold libraries using data from a 5-year retrospective review (*n* =2,696). Filamentous fungi recovered in a routine clinical workflow were put in the context of MALDI-TOF MS reference libraries as a surrogate to predict the applicability of MALDI-TOF MS identification in practice.

Table 1 is a complete list of reported molds from our laboratory and indicates if said molds were included in the Bruker or VITEK spectra databases. Overall, most mold identifications were found in both databases (Fig. 2). Using a genus-level identification specificity or better, the Bruker and VITEK MS libraries contained 80.9 and 83.5%, respectively, of reported mold isolates (88.6 and 91.5% of SOC identifiable molds in the respective libraries). It is impossible to know if the actual species was in the databases for some molds due to the lack of species-level identification used by conventional identification methods. Thus, Table 1 has a “P*” classification to indicate if additional species-level distinction may have been possible. If the databases were consolidated, theoretically 87.1% of molds would have been identified or potentially have had an improved identification. Of note, at the time this study was conducted, the Bruker database did not include the clinically significant thermally dimorphic fungi, such as *Histoplasma*, *Blastomyces*, and *Coccidioides*. Another potential identification improvement with MALDI-TOF MS is improving organism identification specificity beyond “Mold” for the 8.7% of filamentous fungi that were identified by SOC.

### Comparison of Bruker-based extraction methods

The extraction step prior to running MALDI-TOF MS is critical for successful mold identification. Both MALDI-TOF MS systems have recommended mold extraction procedures, but other extractions methods have been reported with varying success (4, 5, 7–10). For example, there is a bead-beating alternative procedure for the Bruker system colloquially known as “the NIH method,” which has been shown to outperform the original Bruker method (5). The NIH group later published a modified NIH (mNIH) extraction method that decreased processing time while also improving identification scores and reproducibility compared to their original method (6). To confirm these findings in preparation for our large head-to-head comparison, two Bruker-based extraction methods (original Bruker and mNIH methods) were tested pairwise in parallel using 24 consecutive clinical isolates identified by conventional mold identification (Table S1). Scores above 95% on the VITEK and 2.0 on the Bruker are widely accepted thresholds for a high confidence identification. Using these criteria, the mNIH extraction method identified more isolates (16 and 8) compared to the Bruker method (14 and 3) using the VITEK and BioTyper systems, respectively (Table 2). Because lowering the BioTyper reporting threshold score has been reported previously (1, 11–14), two additional thresholds are shown in Table 2. Regardless of the thresholds, more identifications were observed using the VITEK system compared to the Bruker system using either extraction method.

**TABLE 2:** Comparison of the original Bruker and mNIH extraction methods (*n* = 24)

Identification confidence scoring (BioTyper/VITEK MS)	BioTyper		VITEK MS	
	Bruker Ext *n* (%)	mNIH Ext *n* (%)	Bruker Ext *n* (%)	mNIH Ext *n* (%)
>2.0/>95%	3 (13)	8 (33)	14 (58)	16 (67)
1.7–2.0	5 (21)	4 (17)	n/a^*a*^	n/a^*a*^
1.5–1.69	3 (13)	1 (4)	n/a^*a*^	n/a^*a*^
≥1.5/>95%	11 (46)	13 (54)	14 (58)	16 (67)
Peaks but no ID	11(46)	8 (33)	4 (17)	2 (8)
No peaks	2 (8)	3 (13)	6 (25)	6 (25)

^*a*^ n/a, not applicable.

Non-identification quality metrics include “Peaks but no ID” and “No peaks.” The former can be the result of poor spectra quality or database insufficiency. This category demonstrated an indirect correlation with overall successful identification rates by extraction method and MALDI-TOF MS system. “No peaks” was less common on the Bruker BioTyper than the VITEK MS for both extraction methods.

### Comparison of extraction methods and MALDI-TOF MS systems

Our primary goal was to compare identification rates of each MALDI-TOF MS system and extraction method using expected clinical mycology workflows. Timely reporting of diagnostic testing is important, and this is especially important for MALDI-TOF MS identification if it is to displace SOC phenotypic macroscopic and microscopic identification. Therefore, our study used early growth (<3 days after visible subcultured growth) to perform a MALDI-TOF MS analysis for 205 consecutive clinical isolates. Isolates were subject to two extraction methods (mNIH and VITEK) for comparison, and both extractions were run on each MALDI-TOF MS system (BioTyper and VITEK MS).

We found the mNIH extraction method resulted in similar identification rates on both MALDI-TOF MS systems (VITEK 56% and BioTyper 54%) when the reporting threshold for the Bruker system was lowered to 1.5 (Table 3, odds ratio 1.06 with a *p*-value of 0.77). The VITEK extraction method had a higher identification rate on the VITEK MS (65%) than the BioTyper (59%) when the reporting threshold for the Bruker system was lowered to 1.5; however, this was not found to be statistically significant (Table 3, odds ratio 1.28 with a *p*-value of 0.22). Of note, having fewer “No peaks” by the Bruker BioTyper is likely the result of manual spectra acquisition being reflexed following automated spectra acquisition failure. Lastly, there was no significant difference in extraction method (mNIH 54% and VITEK 59%) on the Bruker BioTyper using a 1.5 threshold (odds ratio 0.80 with a *p*-value of 0.27). Statistical significance was found when comparing the mNIH extraction method (56%) to the VITEK extraction method (65%) on the VITEK MS (odds ratio 0.66 with a *p*-value of 0.04).

**TABLE 3:** Comparison of the BioTyper and VITEK MS systems using the extraction methods mNIH and VITEK (*n* = 205)

Identification confidence scoring (BioTyper/VITEK MS)	BioTyper		VITEK MS	
	mNIH Ext *n* (%)	VITEK Ext *n* (%)	mNIH Ext *n* (%)	VITEK Ext *n* (%)
>2.0/>95%	47 (23)	68 (33)	114 (56)	134 (65)
1.7–2.0	44 (21)	44 (21)	n/a^*a*^	n/a^*a*^
1.5–1.69	20 (10)	10 (5)	n/a^*a*^	n/a^*a*^
≥1.5/>95%	111 (54)	122 (60)	114 (56)	134 (65)
Discrepant	4 (2)	2 (1)	6 (3)	4 (2)
Peaks but no ID	40 (20)	49 (24)	42 (20)	42 (20)
No peaks	50 (24)	32 (16)	43 (21)	25 (12)

^*a*^ n/a, not applicable.

### Assessment of discrepant and inconsistent findings

Only 65 of the 205 clinical isolates were identified to the same level (63 species- or two genus-level) across Bruker, VITEK, and SOC (Table 4). The major contributing factor (*n* = 89) was the inability of SOC to provide a species-level identification (i.e., insufficient resolution of the reference method). Nine isolates failed to be identified by any of the three methods. Twenty-seven isolates were identified by SOC only. Discrepant results occurred for 13 isolates. To improve our reference method and resolve discrepant findings, non-consensus isolates (*n* = 140) were sent for molecular identification (Table 4). Unfortunately, 24 isolates were unable to be sequenced due to non-viability.

**TABLE 4:** Summary of the discrepant and inconsistent findings across Bruker, VITEK, and SOC approaches (*n* = 205)

Outcomes of identification method comparison	*n* (%)	Sent for molecular identification	Reason	Successful molecular identification *n* (%)
All three methods agree	65 (32)	No	n/a^*a*^ (no additional testing needed)	n/a^*a*^
All three methods failed	9 (4)	Yes	Reference identification needed	5 (2)
SOC lacks species-level identification	89 (43)	Yes	Improve resolution of reference method	78 (38)
SOC identified but MALDIs failed	27 (13)	Yes	Confirm reference method	20 (10)
Discrepant findings	13 (6)	Yes	Resolve discrepant findings	11 (5)
MALDI identified but SOC failed	2 (1)	Yes	Reference needed	2 (1)

^*a*^ n/a, not applicable.

Table 5 shows how each MALDI-TOF MS method and SOC performed using molecular identification as the reference method. SOC identified all but 15 isolates; however, three other isolates that were identified by SOC were found to be misidentified. For the vast majority of isolates (92%), SOC was only able to identify to the genus level, re-illustrating the lack of identification resolution/specificity for traditional phenotypic macroscopic and microscopic identification. When species-level identification was reported, only 50% of isolates were identified accurately.

**TABLE 5:** Reporting accuracy of SOC and MALDI-TOF MS systems using molecular as reference for the 116 non-consensus identifications^*a*^

Outcome	SOC *n* (%)	Bruker BioTyper		VITEK MS	
		mNIH Ext *n* (%)	VITEK Ext *n* (%)	mNIH Ext *n* (%)	VITEK Ext *n* (%)
No ID	15 (13)	60 (52)	55 (47)	56 (48)	47 (41)
Genus discrepancy	3 (3)	1 (1)	1 (1)	1 (1)	0 (0)
Genus agreement	98 (84)	55 (47)	60 (52)	59 (51)	69 (59)
Species agreement	3 (3)	43 (37)	42 (36)	53 (46)	60 (52)
Species discrepancy	3 (3)	5 (4)	12 (10)	5 (4)	7 (6)
Species unspecified	92 (79)	7 (6)	6 (5)	1 (1)	2 (2)

^*a*^ Molecular reference method. Ext: extraction.

The majority of discrepant isolates were not identified using MALDI-TOF MS (Table 5, see “No ID” row). MALDI-TOF MS methods rarely (≤1 per extraction and method combination) had genus-level discrepancies (Table 5) after adjusting for taxonomic updates. Moreover, all MALDI-TOF MS approaches significantly outperformed SOC at species-level identification, as defined by overall successful identifications and species agree-to-discrepant ratios. Importantly, all methods failed to identify a subset of isolates, and all methods had discrepant species-level results (Table 6). Therefore, consolidation of identification methodologies would identify the most molds with the greatest specificity.

**TABLE 6:** Discrepant identifications (in bold) by method

Molecular identification results and comments	SOC	Bruker BioTyper mNIH Ext	Bruker BioTyper VITEK Ext	VITEK MS mNIH Ext	VITEK MS VITEK Ext	Discrepancy comments
*Aspergillus calidoustus* (section *Usti*)	*Aspergillus* species	None	***Aspergillus*** ***ustus***	*Aspergillus calidoustus/ustus*	*Aspergillus calidoustus/ustus*	*A. calidoustus*, a member of *Aspergillus* section *Usti*, is able to grow at 37°C and causes invasive disease in humans. *A. ustus* is unable to grow at 37°C and is very rarely associated with infections in humans (15).
*Aspergillus pseudonomiae* (section *Flavi*)	***Aspergillus flavus***	None	None	None	None	SOC misidentification
*Aspergillus pseudonomiae* (section *Flavi*)	*Aspergillus species*	***Aspergillus nomius***	***Aspergillus nomius***	None	***Aspergillus*** **(mixed species**)	*A. pseudonomiae* and *A. nomius* (orthographic variant of *A. nomiae*) are closely related species that are both members of *Aspergillus* section *Flavi,* falling within the *A. nomius* clade (16).
*Aspergillus sydowii* (series *Versicolores* section *Nidulantes*)	*Aspergillus species*	***Aspergillus versicolor***	None	*Aspergillus* *sydowii*	*Aspergillus* *sydowii*	*A. sydowii* and *A. versicolor* are closely related species within *Aspergillus* series *Versicolores* section *Nidulantes*. *A. sydowii* has reduced susceptibility to amphotericin B and itraconazole (17, 18).
*Aspergillus sydowii* (series *Versicolores* section *Nidulantes*)	*Aspergillus species*	None	***Aspergillus versicolor***	*Aspergillus sydowii*	*Aspergillus sydowii*	
*Aspergillus sydowii* (series *Versicolores* section *Nidulantes*)	*Aspergillus species*	None	***Aspergillus versicolor***	*Aspergillus sydowii*	*Aspergillus sydowii*	
*Aspergillus sydowii* (series *Versicolores* section *Nidulantes*)	*Aspergillus species*	***Aspergillus versicolor***	None	*Aspergillus sydowii*	*Aspergillus sydowii*	
*Aspergillus udagawae* (section *Fumigati*)	***Aspergillus fumigatus***	None	None	***Aspergillus thermomutatus***	***Aspergillus thermomutatus***	*A. udagawae*, *A. fumigatus*, and *A. thermomutatus* are related species within *Aspergillus* section *Fumigati. A. fumigatus* and *A. thermomutatus* are capable of growth at 50°C (18, 19). *A. udagawae* has reduced susceptibility to amphotericin B and azoles, and *A. thermomutatus* has reduced susceptibility to itraconazole.
*Aspergillus westerdijkiae* (section *Circumdati*)	***Aspergillus flavus***	None	None	None	None	SOC misidentification
*Aspergillus westerdijkiae* (section *Circumdati*)	*Aspergillus* species	None	***Aspergillus ochraceus***	None	None	*Aspergillus westerdijkiae* and *A. ochraceus* are both members of *Aspergillus* section *Circumdati* (20).
*Aureobasidium leucospermi*	None	None	***Aureobasidium melanogenum***	None	***Aureobasidium pullulans***	*A. leucospermi*, *A. melanogenum*, and *A. pullulans* are closely related (21, 22). *A. melanogenum* is able to grow at 35°C, while *A. pullulans* is only able to grow to a maximum temperature of 30°C (23).
*Bjerkandera adusta*	***Arthrographis* species**	***Thanatephorus cucumeris***	***Thanatephorus cucumeris***	*Bjerkandera adusta*	*Bjerkandera* *adusta*	*Bjerkandera* spp. produce arthroconidia that can be confused with other arthroconidial-forming species (e.g., *Arthrographis*). The identification of *Bjerkandera adusta* as *Thanatephorus cucumeris* may be attributed to the use of *Bjerkandera* strains misidentified as *Thanatephorus cucumeris* (24).
*Chaetomium species*	None	None	None	***Trichoderma ghanense*** (25)	None	*Chaetomium* species are opportunistic species known to cause rare cases of onychomycosis, skin surface infections, cerebral mycosis, allergies, and eumycetoma (26, 27). *Trichoderma* species are rare causes of infections in humans. When infections occur, they primarily affect the lungs and peritoneum. However, *T. ghanense* has not been associated with human infections.
*Curvularia geniculata*	*Curvularia* species	None	***Curvularia pallescens***	None	None	*C. pallescens* is phylogenetically distant to *C. geniculata* and C. *inequalis*. Identification of these as *C. pallescens* could be attributed to the use of strains in the Bruker database misidentified as *C. pallescens* (28).
*Curvularia inaequalis*	*Curvularia* species	***Curvularia pallescens***	***Curvularia pallescens***	None	None	
*Microascus gracilis*	***Paecilomyces* species**	None	None	None	None	*Microascus gracilis* was originally described as *Paecilomyces fuscatus* based on its subglobose to ellipsoidal conidia. However, this species was found to closely be related to *Scopulariopsis* and redescribed as *Scopulariopsis gracilis*. Following phylogenetic reevaluation of the teleomorph/anamorph taxa, *Microascus* and *Scopulariopsis* showed that some *Microascus* species are phylogenetically distant from some *Scopulariopsis* species. Thus, for some taxa, the *Microascus* name was adopted, and some retained the *Scopulariopsis* name following the unitary nomenclature.
*Mucor velutinosus*	*Mucor* species	***Mucor circinelloides***	***Mucor circinelloides***	*Mucor velutinosus*	*Mucor* *velutinosus*	*M. velutinosus* is a member of the *M. circinelloides* species complex, able to grow at 37°C, and is one of the most clinically important species in this complex. Both *M. velutinosus* and *M. circinneloides* have been observed in blood cultures.
*Parengyodontium album*	***Acremonium* species**	None	None	None	None	*Parengyodontium* species may be mistaken for *Acremonium species* in the formation of conidiogenous cells and conidia. *Parengyodontium* species produce conidia sympodially on denticles, while *Acremonium* species produce conidia blastically on phialides in chains or in slimy heads (29, 30). *Parengyodontium* species have variable susceptibility to the extended spectrum azoles and amphotericin B and reduced susceptibility to the echinocandins. *Acremonium* species generally have *in vitro* resistance to azoles, amphotericin B, and the echinocandins (31, 32).
*Penicillium rubens*	*Penicillium* species	*Penicillium* species	***Penicillium chrysogenum***	***Penicillium chrysogenum***	***Penicillium chrysogenum***	*P. rubens* is closely related to *P. chrysogenum*. Extensive taxonomic studies of *P. chrysogenum* showed that several strains identified as *P. chrysogenum* were actually *P. rubens*, including Fleming’s penicillin producer strain originally identified as *P. chrysogenum* (33).
*Penicillium rubens*	*Penicillium* species	*Penicillium* species	***Penicillium chrysogenum***	***Penicillium chrysogenum***	***Penicillium chrysogenum***	
*Penicillium rubens*	*Penicillium* species	*Penicillium* species	*Penicillium* species	***Penicillium chrysogenum***	***Penicillium chrysogenum***	
*Penicillium sumatraense*	*Penicillium* species	None	***Pencillium citrinum***	None	None	*P. sumatraense* and *P. citrinum* are members of the *Penicillium* section *Citrina*. Both have similar morphology and are indistinguishable from each other. However, they differ in the following: 1) growth at 37°C*, P. sumatranense* (−), *P. citrinum* (+); 2) soluble yellow diffusing pigment, *P. sumatranense* (−), *P. citrinum* (+) (12).
*Saksenaea trapezispora*	None	None	None	***Saksenaea erthrospora***	***Saksenaea erthrospora***	*S. trapezizpora* is a mucoralean fungus that causes severe human and animal cutaneous mucormycoses in both immunocompetent and immunocompromised hosts. *S. erythrospora* can be differentiated from *S*. *trapezizpora* by its ability to rapidly grow at 37°C and at 42°C, while *S*. *trapezizpora* is unable to grow at 42°C and restricted at 37°C (34).
*Trichophyton tonsurans*	*Trichophyton* species	None	None	None	***Trichophyton interdigitale***	*T. tonsurans* is a member of the *T. mentagrophytes* complex comprising species that are phylogenetically closely related to each other (35).

### Filamentous fungi epidemiology in study compared to Pre-COVID-19 pandemic

Our study was performed during the beginning of the coronavirus disease 2019 (COVID-19) pandemic. It is theoretically possible our isolate collection/species prevalence may not reflect a typical year given changes in mask wearing, respiratory illness epidemiology, superinfections following viral illness, isolation, and other patient behaviors (e.g., reluctance to seek healthcare). Therefore, we compared the fungal epidemiology of our 5-year retrospective review and that of the present study. As shown in Table 7, identification rates were largely similar. Only *Penicillium* species reported had a statistically significant decreased identification rate. Increased prevalence was statistically significant for most *Aspergillus fumigatus, Aspergillus flavus*, *Aspergillus niger*, *Paecilomyces* species, and *Scedosporium* species. Other identifications are not shown in Table 7 because their infrequent recovery limited statistical significance calculations.

**TABLE 7:** Fungal epidemiology pre-study and during study

Identifications	Pre-study (%) *n* = 2,696	Study (%) *n* = 205	*p*-value
*Penicillium* species	19.7	12.7	0.017
*Aspergillus fumigatus*	8.8	18.0	<0.0001
*Aspergillus* species (other than listed)	8.8	8.3	NS^*a*^
Mold (nonsporulating/sterile)	8.8	4.9	NS
*Trichophyton* species	8.2	5.4	NS
*Fusarium* species	3.9	6.3	NS
*Cladosporium* species	3.3	2.0	NS
*Aspergillus flavus*	3.1	6.8	0.0051
*Aspergillus niger*	2.8	5.9	0.014
*Alternaria* species	2.6	1.5	NS
*Paecilomyces* species	2.4	4.9	0.036
*Histoplasma capsulatum*	2.4	1.0	NS
*Scedosporium* species	2.2	4.9	0.018
Dematiaceous fungus	2.2	1.0	NS
*Curvularia* species	2.1	1.0	NS
*Aspergillus terreus*	1.2	2.4	NS
*Arthrographis* species	1.2	0.5	NS
*Acremonium* species	1.2	1.0	NS
*Mucor* species	1.1	1.5	NS
*Blastomyces dermatitidis/gilchristii*	1.0	0.5	NS

^*a*^ NS: not significant.

## DISCUSSION

Filamentous fungi are becoming an increasingly important cause of infection, especially in the context of expanding patient populations with complex medical conditions and reduced immune function. Identifying molds accurately and quickly is key to improving patient outcomes. Unfortunately, much of clinical mycology still relies upon conventional phenotypic identification, which may take days to weeks to mature sufficiently after initial visible colony growth and infrequently allows for species-level identification. The main goal of this study was to evaluate the value of MALDI-TOF MS for early clinical mold identification workflows by optimizing current MALDI-TOF MS databases, systems, and extraction methods (Fig. 1). We found that both databases have the majority of reported molds; both systems performed similarly statistically; and the VITEK extraction only outperformed the NIH extraction method on the VITEK system. Taken together, our findings support using MALDI-TOF MS for routine clinical mold identification.

The major benefit of mold identification by MALDI-TOF MS is species-level accuracy, as demonstrated here. In bacteriology, we have learned that high-resolution species-level identification with MALDI-TOF MS can lead to discovering clinical associations and/or unique antimicrobial resistance profiles that went unnoticed with traditional identification methods. Once clinical laboratories routinely report molds to the species-level, species-specific disease associations and antifungal patterns will likely arise. Other potential benefits of MALDI-TOF MS are the low cost per identification, reduced training period for laboratory staff, improved turnaround times when compared to send-out molecular testing, and lower risk of laboratory-acquired infections with faster identifications in colony maturation.

Successful mold identification by MALDI-TOF MS depends on the database, system, extraction method, and colony age. We compared the list of molds from our laboratory to each MALDI-TOF MS database. While, overall, the databases had similar coverage, it is worth noting the absence of thermally dimorphic fungi in the Bruker database. Clinical laboratories considering MALDI-TOF MS for mold identification need to evaluate their own local epidemiology to manage performance expectations. Identification rates will continue to improve, similar to bacteriology, as mold databases are updated. This study also determined that successful mold identification was less dependent on the specific MALDI-TOF MS system and extraction methods. Laboratories should consider system availability and ease of workflow implementation. Given staffing shortages and the need for minimal hands-on time, the extraction methods cannot be laborious and low-throughput. Performing mold extractions and target spotting requires expertise, and success can vary by laboratory (2).

Taxonomic changes create significant challenges for laboratories and clinicians. This is particularly relevant to the rapidly evolving fungal nomenclature. A major challenge facing laboratories (and a challenge in this study) is harmonizing organism names that may appear differently across different identification methods. It is critical that laboratories continue to work with clinical colleagues on taxonomic updates to minimize confusion.

The COVID-19 pandemic impacted healthcare in ways only now being discovered. Since our study was performed largely in the first year of the pandemic, there was concern that our study may not accurately reflect a typical year. One noticeable difference was a decreased volume in fungal cultures compared to previous years. While the rates of different molds overall were fairly consistent, a noticeable drop during the pandemic was observed for *Penicillium* species, a common contaminant. The cause of the decrease is unknown, but it is worth noting that laboratory practices (analytical and post-analytical stages) remained unchanged (i.e., a biosafety cabinet was used for specimen processing and culture workup). A possible explanation is pre-analytical factors, such as different patient populations, decreased surgical specimens, or clinical staff wearing masks. These findings may support a not insignificant portion of contaminants originating prior to specimen receipt.

A major strength of this work is that we directly compared the performance of MALDI-TOF MS instrumentation systems and extraction methods pairwise in the same laboratory. Another strength is that we investigated >200 clinical mold isolates to better reflect clinical performance instead of a challenge mold set that can be biased toward rarely encountered species. Our study prioritized assessing performance under typical clinical laboratories conditions and workflows by testing visible growth ≤3 days and not overly mature colonies, which could be identified using conventional method identification methods. This aggressive identification timeline is reflected in our overall lower identification rates compared to other reports (4, 5, 9, 36, 37). We anticipate continued incubation length improvement as laboratory staff develop a “feel” for choosing the right time to send growth to MALDI-TOF MS. Importantly, this is no different in bacteriology; however, the consequences of delaying identification due to premature colony usage for MALDI-TOF MS are more pronounced. An alternative approach to improving identification rates is to use growth from liquid media. This is a reasonable reflex method, but the turnaround time is more similar to molecular sendout testing.

A limitation of our study was that it was a single center study, and results may not be generalizable. In addition, identification rates can vary by the colony mold mass selected for testing, technologist expertise, and instrument/database updates. It is important that laboratories review these considerations, as well as implement safety precautions to prevent mold spore contamination and accidental staff exposure prior to implementing mold identification by MALDI-TOF MS.

In conclusion, our findings support using MALDI-TOF MS for routine mold identification in clinical laboratories. How MALDI-TOF MS is incorporated into laboratory workflows will vary by laboratory, but we found that a combination of conventional phenotypic identification and MALDI-TOF MS offers early identification with high specificity.

## SUPPLEMENTAL MATERIAL

Table S1 jcm.01548-24-s0001.pdf Isolate list for Table 2.

ASM does not own the copyrights to Supplemental Material that may be linked to, or accessed through, an article. The authors have granted ASM a non-exclusive, world-wide license to publish the Supplemental Material files. Please contact the corresponding author directly for reuse.
