# Supplementary material for: Evaluation of two MALDI-TOF MS systems and extraction methods for identification of filamentous fungi recovered from clinical specimens
Source: J Clin Microbiol. 2025 Jan 14;63(2):e01548-24. doi: 10.1128/jcm.01548-24 (PMC11837564; doi:10.1128/jcm.01548-24)
Supplement: Table S1 — Isolate list for Table 2. [file jcm.01548-24-s0001.pdf]

# Evaluation of Two MALDI-TOF MS Systems and Extraction Methods for Identification of Filamentous Fungi Recovered from Clinical Specimens

Eric M. Ransom[1,2*], Meghan A. Wallace[2], Nathan P. Wiederhold[3], Connie Cañete-Gibas[3], and Carey-Ann D. Burnham[2,4]

[1]Department of Pathology, Case Western Reserve University School of Medicine, Cleveland, Ohio, USA.

[2]Department of Pathology and Immunology, Washington University School of Medicine, St. Louis, Missouri, USA.

[3]Fungus Testing Laboratory, Department of Pathology and Laboratory Medicine, University of Texas Health Science Center at San Antonio, San Antonio, TX 78229, USA.

[4]Department of Molecular Microbiology, Pediatrics, and Medicine, Washington University School of Medicine, St. Louis, Missouri, USA.

Table S1: Isolate list for Table 2

| Organism | *n* |
|---|---|
| *Aspergillus fumigatus* | 5 |
| *Penicillium* species | 5 |
| *Fusarium* species | 3 |
| *Alternaria* species | 2 |
| *Aspergillus* species, not *A. fumigatus* or *A. flavus* | 2 |
| *Curvularia* species | 1 |
| *Cladosporium* species | 1 |
| *Blastomyces dermatitidis/gilchristii* | 1 |
| *Trichophyton* species | 1 |
| *Scedosporium* species | 1 |
| *Paecilomyces* species | 1 |
| *Histoplasma capsulatum* | 1 |
